# Application of Improved YOLOv8 Image Model in Urban Manhole Cover Defect Management and Detection: Case Study

**DOI:** 10.3390/s25134144

**Published:** 2025-07-03

**Authors:** Yanqiong Ding, Baojiang Han, Hua Jiang, Hao Hu, Lei Xue, Jiasen Weng, Zhili Tang, Yuzhang Liu

**Affiliations:** 1 Beijing Information Infrastructure Construction Co., Ltd., Beijing 100080, China; 2 School of Mechanics and Civil Engineering, China University of Mining and Technology, Beijing 100083, China

**Keywords:** manhole cover, model improvement, lightweight network, attention module, multi-scale feature fusion

## Abstract

Manhole covers are crucial for maintaining urban operations and ensuring residents’ travel. The traditional inspection and maintenance management system based on manual judgment has low efficiency and poor accuracy, making it difficult to adapt to the rapidly expanding urban construction and complex environment of manhole covers. To address these challenges, an intelligent management model based on the improved YOLOv8 model is proposed for three types of urban high-frequency defects: “breakage, loss and shift”. We design a lightweight dual-stream feature extraction network and use EfficientNetV2 as the backbone. By introducing the fused MBConv structure, the computational complexity is significantly reduced, while the efficiency of feature extraction is improved. An innovative foreground attention module is introduced to adaptively enhance the features of manhole cover defects, improving the model’s ability to identify defects of various scales. In addition, an optimized feature fusion architecture is constructed by integrating NAS-FPN modules. This structure utilizes bidirectional feature transfer and automatic structure search, significantly enhancing the expressiveness of multi-scale features. A combined loss function design using GIoU loss, dynamically weighted BCE loss, and Distribution Focal Loss (DFL) is adopted to address the issues of sample imbalance and inter-class differences. The experimental results show that the model achieved excellent performance in multiple indicators of manhole cover defect recognition, especially in classification accuracy, recall rate, and F1-score, with an overall recognition accuracy of 98.6%. The application of the improved model in the new smart management system for urban manhole covers can significantly improve management efficiency.

## 1. Introduction

With the acceleration of global urbanization, the construction of smart cities has become an important means to enhance urban management and improve the quality of life for residents. In the development of smart cities, the safe operation of urban infrastructure is crucial. As a critical component of urban infrastructure, the safety of manhole covers is directly related to urban operations and the safety of residents’ travel. According to statistics, safety incidents caused by manhole cover defects occur frequently each year, posing significant challenges to urban management and inconveniences to residents’ lives. Therefore, how to efficiently and accurately detect manhole cover defects has become an urgent issue for urban management authorities.

Traditional methods for detecting manhole cover defects primarily rely on manual inspections, which are not only inefficient but also limited in coverage, making it difficult to meet the modern city’s demands for real-time and accurate defect detection [1,2]. As a foundational breakthrough in intelligent infrastructure inspection, Hinton and Salakhutdinov pioneered deep autoencoders that learn nonlinear low-dimensional representations through layer-wise pre-training [3]. This work validated the feasibility of deep learning-based computer vision for urban road defect recognition, paving the way for automated manhole cover detection systems. However, existing intelligent recognition methods face challenges such as insufficient feature extraction and poor detection performance for small targets [4] in complex environments, resulting in low detection accuracy and difficulties in meeting practical application requirements. In existing research, although some scholars have attempted to utilize deep learning techniques to address the issue of manhole cover defect detection [5], there are still several limitations. For example, the study by Kong Tianyu and Dai Jiguang [6] proposed an improved YOLOv5-based method for detecting manhole cover defects on road surfaces. However, its feature extraction capability in complex environments is limited, resulting in the insufficient generalization ability of the model in practical applications. Additionally, Chen Jianyu et al. [7] improved the YOLOv5 object detection algorithm to achieve the rapid and intelligent detection of common road surface defects, but the model’s accuracy and environmental adaptability still have significant room for improvement. Meanwhile, Cheng Ying et al. [8] failed to effectively address the issue of sample imbalance in their loss function design, leading to weak recognition ability for minority categories. Furthermore, Huang Wenlong [9] proposed an improved YOLOv5s-based real-time vehicle-mounted image road defect detection model, which can detect multi-scale road defects to some extent. However, the model’s detection scope is limited due to the absence of non-road defect categories in the dataset. Moreover, Xu Mengbing [10] introduced a manhole cover defect detection method combining 3D laser point clouds, which directly and accurately obtains information such as manhole cover locations and defects. Nevertheless, it still cannot identify cases where the manhole cover is incomplete or where the intensity of the manhole cover point cloud is extremely similar to the surrounding road surface threshold. These shortcomings in existing research indicate that current methods still require further improvement in areas such as feature extraction, multi-scale feature fusion, and handling sample imbalance.

To address the aforementioned issues, this paper proposes an improved YOLOv8-based intelligent recognition method for manhole cover defects. The method significantly enhances the model’s feature extraction capability and multi-scale feature representation by introducing a lightweight dual-stream feature extraction network, an innovative foreground attention module, and an optimized feature fusion architecture. Additionally, a hybrid loss function combining GIoU loss, dynamically weighted BCE loss, and Distribution Focal Loss (DFL) [11,12,13,14] is designed to effectively mitigate the impact of sample imbalance and inter-class variations on model performance [15].

## 2. Related Work

YOLO (You Only Look Once), as a representative model of target detection, has been iterating continuously since its advent in 2015 and has become a core technology in the fields of industrial detection, intelligent monitoring, and so on. The YOLO series has always been aiming at more accurate and faster recognition. YOLOv1 first converted target detection into a regression problem through grid division and full connection layer real-time detection. YOLOv2 introduces anchor boxes and batch normalization, optimizes anchor box size through K-means clustering, and significantly improves detection accuracy [16]. YOLOv3 further used multi-scale detection and the residual network Darknet-53 to effectively identify small targets for the first time [17,18]. YOLOv4 integrates the CSPDarknet-53 backbone network, spatial pyramid pooling (SPP), and the path aggregation network PANet to improve robustness in complex scenarios [19]. From YOLOv5 to the PyTorch framework, the first industrial-level solution was introduced [20]. YOLOv6 and v7 further optimize the efficiency of feature fusion by re-parameterizing the backbone EfficientRep and the extension layer aggregation network E-Elan [21].

As a milestone version launched by Ultralytics, YOLOv8 adopts anchor-free design and modular architecture and improves small-target positioning accuracy while maintaining real-time performance by decoupling the detection head and dynamic label assignment [16]. Its hardware-efficient architecture, such as its hybrid scale attention mechanism, makes its reasoning speed on edge devices more than 30% higher than that of v5 [21]. YOLOv9 to v12 further improve accuracy in complex scenes, but the computational cost increases significantly.

Compared with the latest versions such as YOLOv12, YOLOv8 has the following core advantages in the intelligent manhole cover detection problem: Its excellent lightweight and edge adaptability make it more suitable for industrial production and applications and avoid the detection delay caused by insufficient computing power. The free design of the anchor frame of YOLOv8 eliminates the dependence of the traditional anchor frame on the size change in the well cover. Combined with the dynamic label allocation strategy, the missing detection rate in complex scenes such as well cover occlusion and tilt is significantly improved. In contrast, new versions such as v12 have higher adaptation costs for industrial deployment due to their novel architecture and lack the support of mature third-party optimization tools. This study chose YOLOv8 as the basis, not only because of its balanced performance in speed and accuracy but also because of its large amount of practical verification accumulated in industrial scenes such as infrastructure monitoring. Through targeted optimization, YOLOv8 can better adapt to multi-scale, low-light, and other challenges in urban manhole cover defect detection and provide practical technical solutions for the real-time monitoring of intelligent cities.

Although recent advances in computer vision have made significant progress in multi-scale target detection and attention mechanisms for real-time applications, several limitations persist when applied to infrastructure defect detection. Dang et al. [22] proposed HA-FPN, a hierarchical attention feature pyramid network that combines transformer features with channel attention, but their approach suffers from quadratic scaling with feature size and achieves only 12 FPS compared to 18 FPS for the baseline FPN, making real-time infrastructure monitoring challenging. Qiao et al. [23] developed DetectoRS with recursive feature pyramid and switchable atrous convolution, yet their method’s computational overhead grows substantially with each recursive layer, limiting practical scalability for continuous monitoring applications. Dai et al. [24] introduced Dynamic Head that unifies multiple attention mechanisms without theoretical computational cost; however, the unified approach requires significant GPU memory and causes training instability due to complex attention interactions. Tan et al. [25] achieved substantial efficiency improvements with EfficientDet through bidirectional feature pyramid networks, but their method still struggles with very small objects (<32 × 32 pixels) that are critical for detecting early-stage defects in manhole covers. Woo et al. [26] demonstrated improvements with CBAM attention modules, though their approach relies on simple pooling operations that miss complex spatial relationships and requires extensive hyperparameter tuning for each deployment scenario. Liu et al. [27] developed an adaptive image segmentation network specifically for surface defect detection, but their method faces texture similarity challenges between defective and normal areas, making reliable detection of subtle manhole cover deterioration difficult. Guan et al. [28] specifically targeted manhole cover detection with deep learning models, yet their approach shows performance degradation under varying lighting conditions and requires substantial computational resources that limit embedded deployment. These shortcomings in existing research indicate that current methods still require further improvement in areas such as feature extraction, multi-scale feature fusion, and handling sample imbalance.

## 3. Urban Manhole Cover Intelligent Operation and Maintenance Management System

### 3.1. The Dilemma of Traditional Management Modes

According to statistics, there are approximately 3,600,000 water, electricity, gas, communication, and heating utility covers in urban Beijing. With the improvement of urbanization levels, the surge in the number of utility covers has led to a significant increase in the frequency and occurrence of cover defects, imposing huge management pressures on the departments responsible for their maintenance. Unauthorized private connections pose severe threats to the safe operation of urban underground lifeline projects, jeopardizing residents’ daily lives and personal property safety, leading to economic losses in communication line operations.

Early management and maintenance of utility covers relied mainly on manual efforts. Later, respective ownership departments began using computers for management and maintenance, but this was limited to replacing manual paper records with digital ones. Currently, the primary mode of cover management still heavily relies on manual inspection and survey, involving periodic patrols, recording, and repairs. This approach is inefficient, consumes considerable manpower and resources, and easily reaches saturation as the number of covers increases. Traditional inspections have numerous drawbacks that fail to meet current needs, and challenges in cover management primarily include the following aspects:

The efficiency of current cover management is low, with the entire process from ordering inspections to identifying defects requiring human involvement. There is a low degree of defect classification and specificity, urgently necessitating the enhancement of cover management systems through smart upgrades.

The utilization rate of each acquired cover image is low, without establishing a database of cover defects, making it impossible to comprehensively identify defect characteristics across different areas or statistically analyze defect distribution patterns within the city.

The factors influencing cover defects are unclear; many covers in good health condition are prematurely replaced due to human recognition errors, resulting in resource waste.

Given the vast urban landscape and the scattered placement of utility covers across major roads, pedestrian pathways, and residential zones, patrol and inspection operations face challenges with right-of-way management. The frequent stops required during these tasks introduce substantial traffic safety risks.

Based on these problems in cover defect management, this project, relying on BII Company’s management of communication covers in central Beijing, aims to address existing issues in cover management systems. It focuses on the intelligent upgrade of cover management models, covering comprehensive smart upgrades from system architecture to the establishment of defect databases and intelligent recognition systems, aiming to establish a refined and intelligent management system for urban cover defects.

### 3.2. Intelligent Management System Architecture

An intelligent management system for urban manhole cover defects is designed with a four-layer architecture, including a Perceptual Layer, Identification Layer, Warning Layer, and Response Layer, working collaboratively to enable end-to-end smart management of manhole cover defects.

Within the Perceptual Layer, a comprehensive data acquisition system based on the “point–line–face” concept is implemented to collect data on manhole covers, including images of defects. The “Point Supervisory Control System” involves installing monitoring equipment at key locations to capture the real-time health status and environmental conditions of critical manhole covers. “Line: Inspection worker on-site shooting” dynamically adjusts patrol routes according to maintenance frequency and recurring defect patterns, improving inspection efficiency. Meanwhile, “Face: Patrol car automatic extraction” deploys intelligent inspection vehicles to automatically collect road-side manhole cover images, forming a regional image perception network that provides foundational data for further analysis.

In the Identification Layer, image recognition technology is used to learn and identify different types of manhole cover defects. Collected data is uploaded to a cloud platform equipped with pre-configured deep learning algorithms for object detection and image classification. By labeling images according to defect categories, a classification model is trained to enable the automated identification and categorization of manhole cover defects.

**Figure 1:**
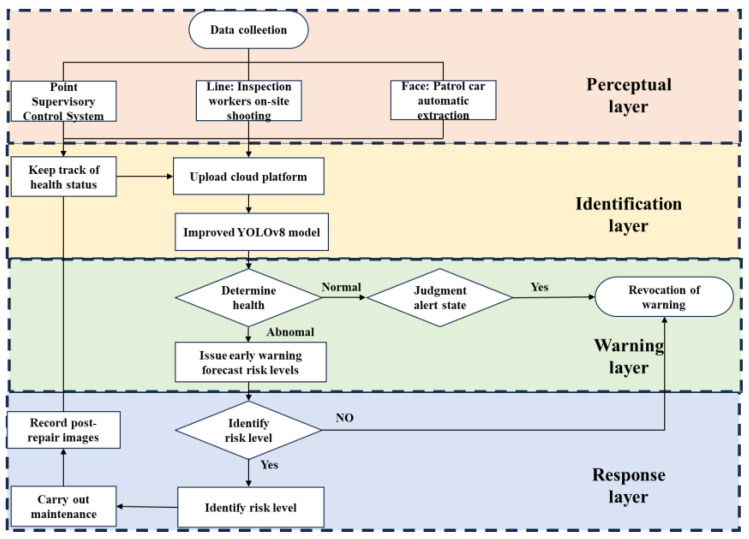
Urban manhole cover maintenance and operation process flowchart.

In the Warning Layer, the system evaluates the identification results. If a manhole cover is determined to be in good condition, no alert is generated. However, if it is classified as sub-healthy, an alert is triggered based on the type of defect, accompanied by a risk level assessment and preliminary maintenance suggestions derived from historical repair records, supporting informed decision-making for subsequent actions, as shown in Figure 1.

Once alerts are passed to the Response Layer, they undergo human review and processing. If the alert is confirmed to be accurate, the associated risk level and maintenance recommendations are accepted. In cases where false positives occur, such as when the cover is actually in good condition, or when risk assessments are inaccurate, manual adjustments are made before finalizing the evaluation. Based on the verified maintenance recommendations, targeted repairs and maintenance are carried out on the corresponding manhole cover structures, thus completing the closed-loop management process.

## 4. The Proposed Method

This paper proposes an improved YOLOv8 model for the classification of manhole cover defect images. By optimizing the backbone network, attention mechanism, feature fusion module, and loss function, the model’s accuracy and generalization capability in identifying manhole cover defects are significantly enhanced. The improved model is capable of more effectively capturing the critical features of manhole cover defects, thereby achieving more accurate classification.

### 4.1. YOLOv8 Network Architecture

**Figure 2:**
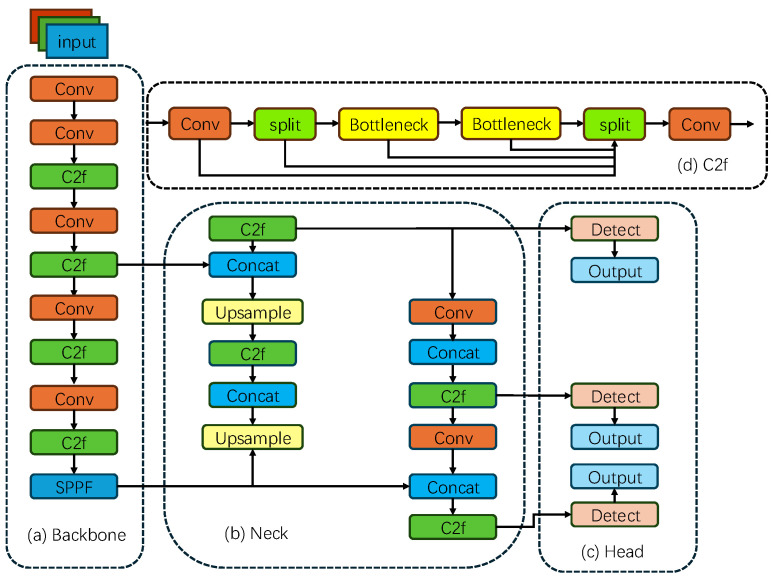
Structure of the YOLOv8 model.

With the advancement of computer vision technology, real-time object detection [29] has found increasingly widespread applications in fields such as autonomous driving and intelligent surveillance. In 2015, Joseph Redmon and his colleagues introduced the YOLO (You Only Look Once) single-stage object detection algorithm, which transformed object detection into a regression problem [30], achieving a breakthrough in real-time detection. However, early YOLO models still exhibited limitations in detecting small objects and localization accuracy. To address these issues, researchers have continuously refined YOLO. In January 2023, to strike a balance between detection accuracy and computational efficiency in complex scenarios, Glenn Jocher and his team at Ultralytics proposed YOLOv8 [31]. This model comprises four main components, including the backbone, neck, head, and loss function, with its architecture illustrated in Figure 2.

The input image is first processed by a series of convolutional layers for feature extraction, followed by further processing through the C2f module [32]. The C2f module replaces the traditional concatenated Bottleneck modules with gradient connections. This design significantly enhances the richness of gradient flow while maintaining the model’s lightweight nature, thereby improving the efficiency and quality of feature extraction. The extracted feature maps are then fused through the neck network, which adopts a feature pyramid network (FPN) structure. Through upsampling and concatenation operations, the neck network effectively integrates feature maps from different levels, expanding the model’s receptive field and enhancing its ability to capture multi-scale features. The head network employs an anchor-free structure, directly predicting the target’s position and size, which avoids the limitations of preset anchor boxes and improves detection accuracy. The loss function combines the Distribution Focal Loss and the CIoU loss as the key components of the regression loss. Additionally, a Task-Aligned Assigner is introduced to optimize sample matching, further enhancing the model’s detection performance.

### 4.2. The Improved YOLOv8 Manhole Cover Defect Detection Model

**Figure 3:**
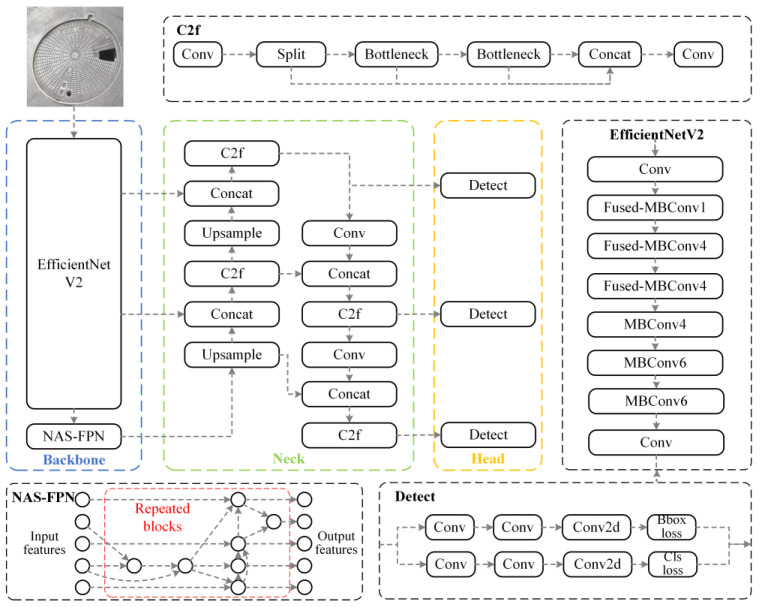
Architecture of the improved YOLOv8 model.

To enhance the performance of the YOLOv8 model in the context of manhole cover defect detection, this study systematically improved its network architecture. By introducing EfficientNetV2 as the backbone network and leveraging its neural architecture search (NAS)-optimized structure and fused MBConv modules [33], the model achieved optimization in terms of parameter count and computational efficiency. Building upon this, a Foreground Attention Enhancement Module (FAEM) based on spatial attention mechanisms was designed to strengthen the model’s ability to perceive and extract features of manhole cover defects. Additionally, the NAS-FPN feature fusion network was integrated to improve multi-scale feature fusion through automated search for optimal feature fusion paths [34]. The improved network architecture is illustrated in Figure 3.

#### 4.2.1. Lightweight Backbone Network

YOLOv8 employs CSPDarknet53 as its backbone network, which, despite its robust feature extraction capabilities, suffers from computational redundancy and low parameter utilization when processing large volumes of manhole cover images. To address this issue, this study introduces EfficientNetV2 as an enhanced backbone network. EfficientNetV2 significantly improves training speed and parameter efficiency while maintaining model lightweightness by combining training-aware neural architecture search with scaling techniques. Specifically, the network incorporates the fused MBConv [35] structure in its early layers, effectively reducing memory access overhead. In the convolutional operations, this structure optimizes the sliding process of convolutional kernels over the input matrix. Namely,

(1) $$O_{i,j}=\sum_{p=0}^{k-1}\sum_{q=0}^{k-1}I_{i+p,j+q}\cdot K_{p,q}$$

EfficientNetV2 tends to use smaller expansion ratios and 3 × 3 convolutional kernels, compensating for the reduction in the receptive field by increasing the number of network layers. Additionally, the network completely removes the last stride-1 stage present in the original EfficientNet, further reducing the number of parameters and memory access overhead. As a result, it significantly outperforms traditional models in terms of training speed and parameter efficiency.

#### 4.2.2. Attention Mechanism

**Figure 4:**
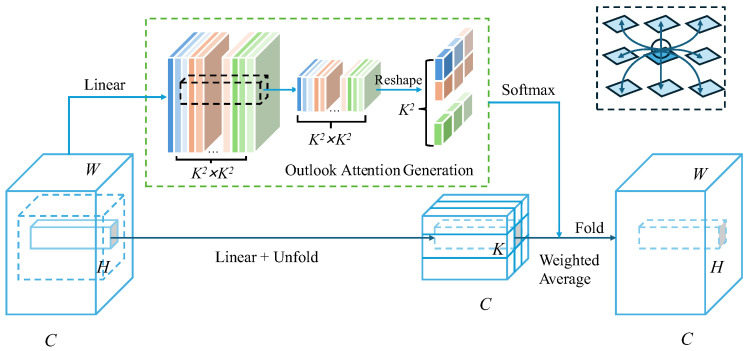
Network structure of the foreground attention mechanism.

Given the presence of interfering factors in street-view images, such as ground text markings, circular traffic signs, and water stains, which can cause feature contamination during the feature extraction process, this paper introduces a foreground attention mechanism (Outlook Attention) to enhance the model’s ability to perceive and extract features of manhole cover defects. As shown in Figure 4, the network structure of the foreground attention mechanism [36] is illustrated. For an input feature map of size C × H × W × F (where C represents the number of channels, H the height, W the width, and F the feature dimensions), the foreground attention mechanism generates a foreground mask to highlight the target foreground regions. This mask is then multiplied element-wise with the input feature map, resulting in a feature map of the size C × H × W × F that incorporates foreground information.

#### 4.2.3. Feature Fusion Optimization

To enhance the model’s ability to fuse multi-scale features, this study optimizes the feature fusion module by adopting the NAS-FPN (neural architecture search feature pyramid network) fusion module [37]. NAS-FPN achieves the automatic optimization of the FPN by exploring the optimal network architecture within a large search space, ensuring performance while reducing network complexity and improving computational efficiency. Specifically, NAS-FPN employs a neural architecture search algorithm to identify the optimal network structure $N^{*}$ within the search space S, maximizing the performance metric L on the given dataset D, as expressed by the following equation:

(2) $$N^{*}=arg\underset{N\in S}{max}L\left(N,D\right)$$

This automated search process effectively avoids the limitations of manually designing feature fusion structures, uncovering feature fusion methods that are better suited to specific tasks. During the feature fusion process, NAS-FPN performs weighted fusion on feature maps from different levels. For example, for the feature maps ${F^{i}\;and\;F}^{i+1}$, the fused feature map $F^{\mathrm{fused}}$ can be expressed as

(3) $$F^{\mathrm{fused}}=w_{1}F^{i}+w_{2}F^{i+1}$$

Here, the weights $w_{1}$ and $w_{2}$ are learned during training and satisfy $w_{1}$ + $w_{2}$ = 1. This dynamic weighted fusion strategy enables the model to automatically allocate the importance of features at different scales based on the specific task requirements, thereby significantly enhancing the model’s ability to capture defect features at various scales. In defect detection tasks, the model can more accurately localize and identify defect regions, reducing missed detections and false positives, and improving overall detection performance metrics such as accuracy and recall. Furthermore, the optimized network structure of NAS-FPN reduces computational complexity while maintaining performance, enhancing computational efficiency and making the model more suitable for real-time detection applications.

### 4.3. Loss Function Design

In the task of manhole cover defect classification, traditional loss functions often fail to effectively address issues such as small inter-class differences, large intra-class variations, sample imbalance, and insufficient robustness in complex scenarios. To tackle these challenges, this paper designs a hybrid loss function that combines GIoU loss, dynamically weighted BCE loss, and Distribution Focal Loss (DFL), significantly improving the model’s classification accuracy and robustness. Specifically, the GIoU loss calculates the generalized intersection over union (IoU) [38] between predicted and ground-truth bounding boxes, addressing the gradient vanishing problem for non-overlapping boxes and enhancing the precision of bounding box regression. This effectively reduces misclassification rates between similar categories, such as “shift” and “loss” defects. The loss function is expressed as Equation (4):

(4) $$L_{GIoU}=1-GIoU$$

The calculation formula for GIoU is given by Equation (5) as follows:

(5) $$GIoU=\frac{\mathrm{Area}\;\mathrm{of}\;\mathrm{overlap}}{\mathrm{Area}\;\mathrm{of}\;\mathrm{Union}}-\frac{\mathrm{Area}\;\mathrm{of}\;\mathrm{Empty}\;\mathrm{Space}}{\mathrm{Area}\;\mathrm{of}\;\mathrm{Convex}\;\mathrm{Hull}}$$

The dynamically weighted BCE loss adjusts weights based on the number of samples in each category, enhancing attention to minority classes and improving classification balance. This effectively addresses the geometric feature differences among various forms within the “damaged” category. The loss function is defined as Equation (6):

(6) $$L_{DBCE}=-\sum_{c=1}^{C}\frac{N_{\mathrm{total}}}{N_{c}}\left[y_{c}logp_{c}+\left(1-y_{c}\right)log\left(1-p_{c}\right)\right]$$

where C is the number of categories, $N_{\mathrm{total}}$ is the total number of samples, $N_{c}$ is the number of samples in the c-th category, $y_{c}$ is the ground-truth label, and $p_{c}$ is the predicted probability by the model.

The Distribution Focal Loss (DFL) models bounding box regression as a probability distribution and optimizes hard sample learning through KL divergence. This enhances the model’s ability to recognize minority classes and addresses the issue of low recall rates for minority categories caused by sample imbalance. The loss function is defined as Equation (7):

(7) $$L_{DFL}=\sum_{i=1}^{M}D_{KL}\left(P_{\mathrm{pred}}∥P_{\mathrm{true}}\right)$$

where M is the dimensionality of the bounding box, $P_{\mathrm{pred}}$ is the predicted probability distribution, $P_{\mathrm{true}}$ is the true target distribution, and $D_{KL}$ is the KL divergence.

By integrating these three loss functions through weighted combination, a comprehensive hybrid loss function is formed. This not only enhances the model’s robustness in complex scenarios but also reduces computational overhead by simplifying the model structure and optimizing the loss function. As a result, the model’s operational efficiency on edge devices is improved, meeting the real-time requirements of municipal inspections [39]. The hybrid loss function is defined as Equation (8):

(8) $$L_{\mathrm{total}}=\alpha L_{GIoU}+\beta L_{DBCE}+\gamma L_{DFL}$$

where α, β, and γ are weighting coefficients that can be adjusted based on practical requirements. The recommended weight settings are the following: α = 0.5; β = 0.3; and γ = 0.2.

## 5. Experimental Design

### 5.1. Dataset Construction

#### 5.1.1. Data Acquisition

In this study, the image data sources of well cover defects were mainly accumulated by BII Company in the process of manual inspection and maintenance during the operation and maintenance management of well cover in the six districts of Beijing in the past five years. This paper involves three types of well cover defects, “breakage, loss, and shift”, and completely defect-free well covers classified as “good”. Breakage, loss, and shift were obtained through manual image acquisition during inspection, and the good category was obtained after the maintenance engineer reported the maintenance effect. During the data labeling inspection, this paper labeled the defects in strict accordance with the types of defects found in the inspection and confirmed the categorization of some defects that were difficult to distinguish. The blurred pictures were taken by communicating with the field engineer to ensure the accuracy of the data labeling, which provided the basis for the later intelligent recognition training. This dataset covers well covers of different environments in the six districts of the city, such as well cover images of sidewalks and main roads, day and night, traffic intersections, parking lots, and other environmental scenes. The environment is comprehensive.

**Figure 5:**
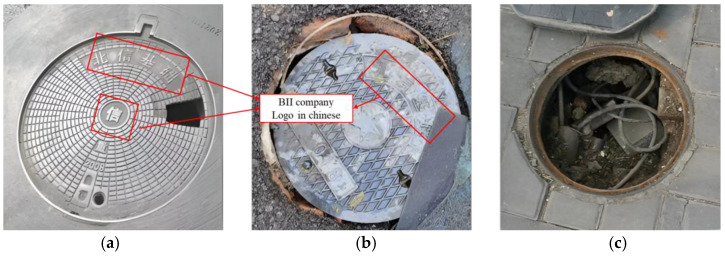
Illustration of three types of defects: (**a**) breakage; (**b**) shift; (**c**) loss.

Figure 5 illustrates the three primary types of defects. To enhance the accuracy and robustness of the defect classification model proposed in this paper, the dataset was carefully curated to include images affected by varying lighting angles, different levels of clarity, and various types of stains. The selected images were cropped to highlight the target areas and resized to a resolution of 512 × 512 pixels. Damage to the manhole cover itself, such as cracks or gaps, as well as damage around the manhole, was categorized as “breakage”. Cases where the manhole cover had shifted, exposing the manhole opening, were classified as “shift”. Instances where the manhole cover was completely missing or displaced, leaving the hole fully exposed, were labeled as “loss”.

**Figure 6:**
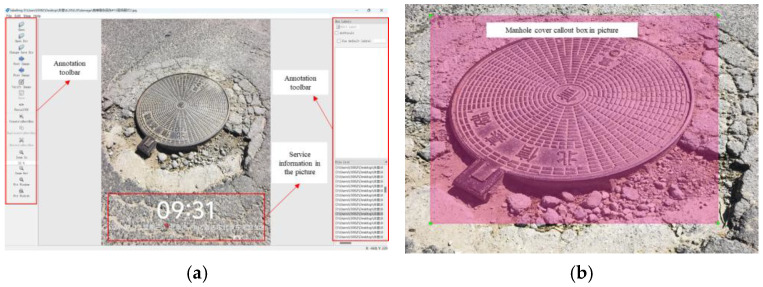
Image annotation: (**a**) Labeling software interface. (**b**) Annotation results.

For the manhole cover defect image classification dataset, all images were manually annotated, with 80% of the images allocated to the training set and 20% to the test set. The labeling software was used to mark the defective areas of each manhole cover image. Figure 6 shows the interface of the labeling software when annotating a damaged manhole cover image, as well as the annotation results for a damaged manhole cover image. Each annotated image has a corresponding TXT file, as illustrated in Figure 6.

#### 5.1.2. Data Analysis

**Figure 7:**
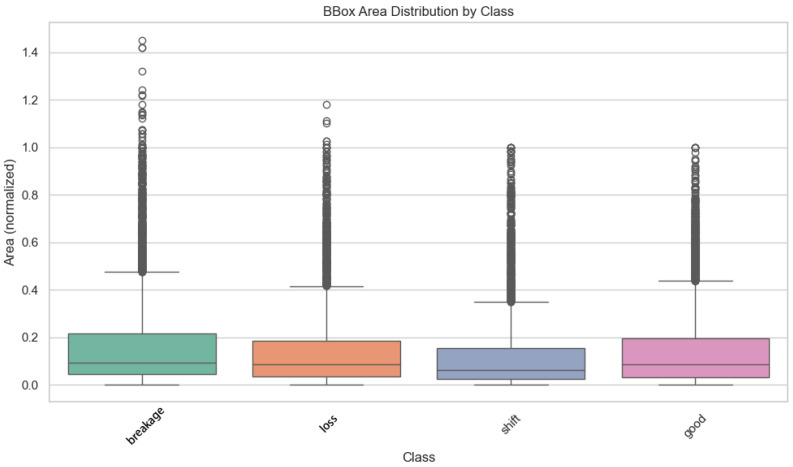
Distribution of labeled areas for each category.

**Figure 8:**
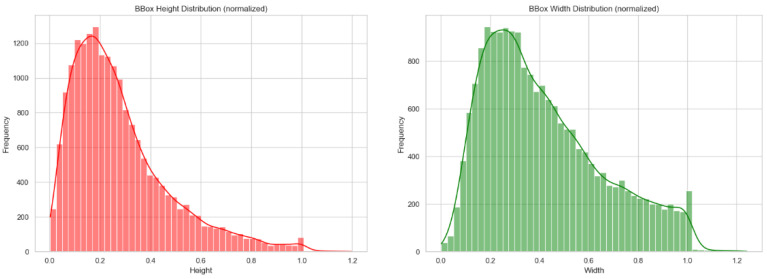
Size distribution of manhole cover images.

The dataset exhibits substantial scale variations, which pose a significant challenge for multi-scale feature detection. The normalized distribution of bounding box areas (Figure 7) indicates that small targets dominate, primarily concentrated in the 0.1–0.4 range. Specifically, the “breakage”, “loss”, and “shift” categories show large interquartile ranges and outliers, reflecting substantial variations in the spatial extent of “breakage”, “loss”, and “shift” defects. In contrast, the “good” category demonstrates more consistent size stability. The normalized distributions of bounding box height and width (Figure 8) further confirm these scale variations. Both height and width exhibit right-skewed distributions, with a few large-sized targets in the tail (e.g., significant variations in the aspect ratio of the “loss” category) contrasting sharply with the high-frequency small targets. These scale variations necessitate the enhancement of multi-scale feature pyramids and attention mechanisms to improve small-target detection and shape adaptability.

**Figure 9:**
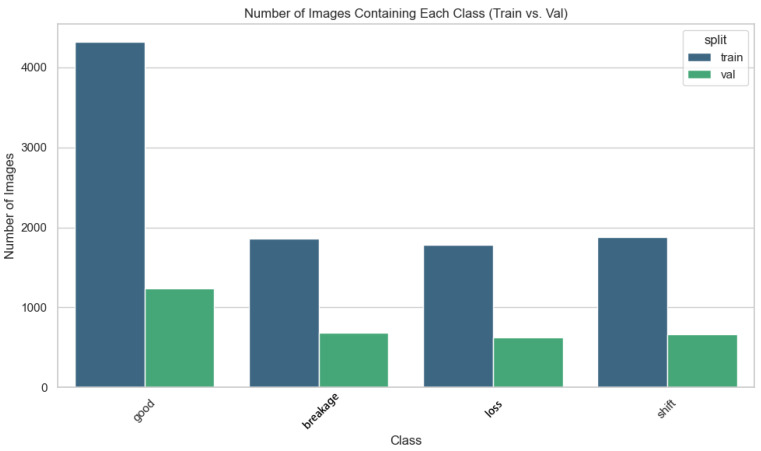
Classification of training set and test set in dataset.

The dataset also suffers from severe class imbalance, which can impair the model’s ability to recognize minority classes. Figure 9 illustrates the class distribution, showing that the number of samples in the “good” category far exceeds those in the other three categories. This imbalance may lead to the inadequate recognition of minority classes such as “breakage”, “loss”, and “shift”. To address this issue, data augmentation and resampling strategies should be employed to balance the class distribution, ensuring that the model can effectively learn the features of all classes.

**Figure 10:**
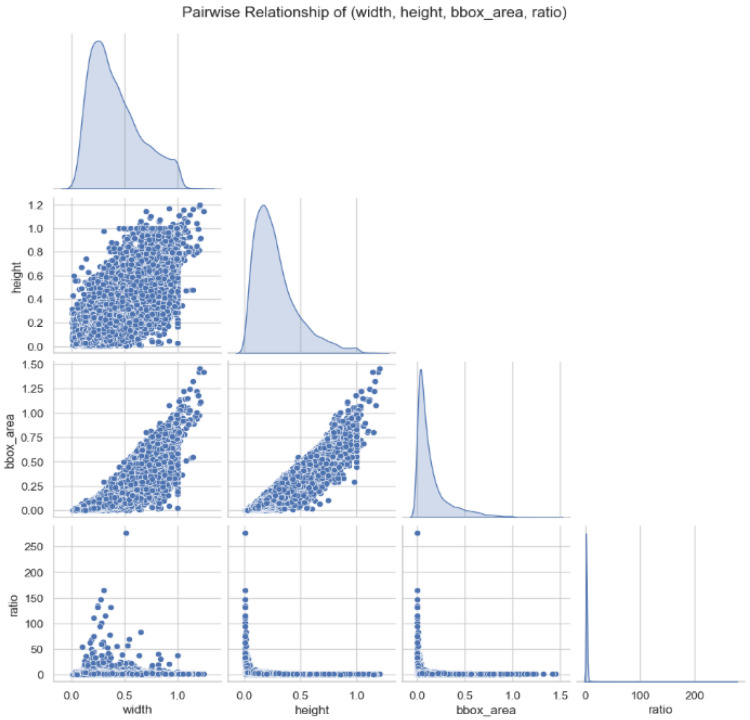
Visualization of multivariate relationships.

Multivariate analysis (Figure 10) reveals complex relationships among the geometric characteristics of the targets. Width and height exhibit a positive correlation but include shape outliers, while area increases linearly with size. The dispersed distribution of aspect ratios, particularly the significant variations in the “loss” category, highlights the inherent differences in morphological features among different defect types. These findings suggest that model optimization should focus on enhancing multi-scale feature pyramids and attention mechanisms to improve small-target detection and shape adaptability. Additionally, data augmentation and resampling strategies should be employed to balance class distribution, and a learning framework that integrates spatial information and morphological features should be constructed to address the core challenges of large-scale variations, significant shape diversity, and sample imbalance in manhole cover defect detection.

#### 5.1.3. Data Augmentation

**Figure 11:**
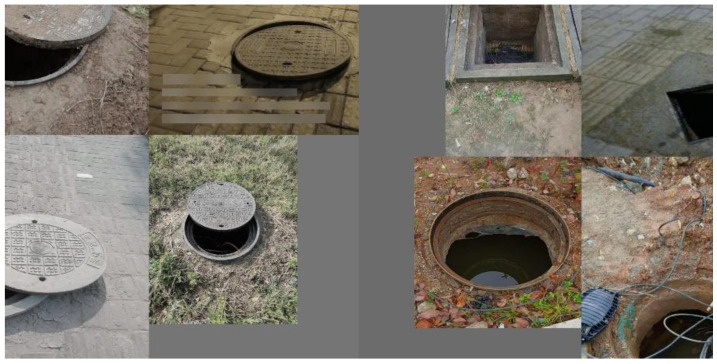
Image enhancement based on mosaic enhancement technique.

In the task of manhole cover defect recognition, variations in image acquisition conditions often introduce changes in angle, scale, and lighting. To enhance the model’s robustness and generalization ability, this paper adopts a comprehensive data augmentation strategy. Geometric transformation methods include random rotation (−15° to +15°) to simulate different shooting perspectives, while random cropping reduces background interference, allowing the model to focus more on defect regions. Scaling operations address multi-scale challenges by adjusting target sizes at different resolutions. The Mosaic augmentation technique (as shown in Figure 11) intelligently combines four randomly selected image fragments into a single training sample, further enhancing scene diversity [40]. This enables the model to encounter heterogeneous defect patterns and backgrounds within a single sample, while also improving small-target detection capabilities—particularly crucial for subtle defects such as manhole cover displacement. To mitigate the effects of inconsistent lighting and sensor noise, this paper employs rigorous image normalization: pixel values are linearly normalized to the range [0, 1] using Equation (9).

(9) $$x^{\prime }=\frac{x-\mu }{\sigma }$$

where x is the pixel value of the original image, μ is the mean of the image, σ is the standard deviation of the image, and $x^{\prime }$ is the normalized pixel value. This operation ensures uniform brightness and contrast across images, thereby reducing the impact of lighting variations on model performance. This cascaded augmentation approach collaboratively addresses key challenges in defect recognition, including scale variations, class imbalance, and environmental changes, while maintaining photometric consistency across heterogeneous input data.

Through a series of data augmentation techniques, the final number of images for the four types of manhole cover defects are as follows: “good”, 7515 images; “breakage”, 3991 images; “loss”, 3819 images; and “shift”, 3883 images.

### 5.2. Experimental Setup

#### 5.2.1. Experimental Environment Configuration

To ensure that the training and testing of the manhole cover defect recognition model could be conducted in an efficient computing environment, this experiment was set up based on a high-performance computing platform. The specific hardware and software configurations are shown in Table 1 and Table 2.

In terms of optimizer selection, the AdamW optimizer was employed, which combines weight decay strategies to enhance the model’s generalization capability. The initial learning rate was set to 0.001, and a cosine annealing learning rate schedule was adopted to ensure stable training. Each training iteration was performed with a batch size of 64, with a maximum of 200 training epochs. The input image size was set to 512 × 512 to balance the precision of feature extraction and computational efficiency.

#### 5.2.2. Evaluation Criteria for Experiments

The commonly used accuracy metric for image classification tasks was adopted as the evaluation index. For the task of the multi-classification of manhole cover defect images in this paper, the overall model performance was evaluated using macro-averaging. The metrics for each class were calculated separately, and then the arithmetic mean of these metrics across all classes was computed to obtain the average precision, average recall, and average F1-score. Accuracy (Acc) is the proportion of samples correctly classified by the model out of the total number of samples; precision (P), also known as the positive predictive value [41,42,43], is the proportion of true positive samples among the samples predicted as positive by the model; recall (R), also known as sensitivity, is the proportion of true positive samples among the samples that are actually positive, and a higher value indicates a lower false-negative rate of the model; the F1-score is the harmonic mean of precision and recall, considering both metrics, and a higher value is preferred. The calculation formulas are as follows:

(10) $$Accuracy=\frac{TN+TP}{TP+FP+TN+FN}$$

(11) $$Precision=\frac{TP}{TP+FP}$$

(12) $$Recall=\frac{TP}{TP+FN}$$

(13) $$F1-Score=2*\frac{\mathrm{Precision}*\mathrm{Recall}}{\mathrm{Precision}+\mathrm{Recall}}=\frac{TP*2}{TP*2+FN+FP}$$

In Equations (10)–(13), the following metrics are defined: true positives (TP) represent the number of correctly predicted positive samples; false negatives (FN) denote the number of positive samples incorrectly predicted as negative; false positives (FP) indicate the number of negative samples erroneously predicted as positive; and true negatives (TN) correspond to the number of correctly predicted negative samples [44].

### 5.3. Results and Analysis

#### 5.3.1. Performance Assessment

**Figure 12:**
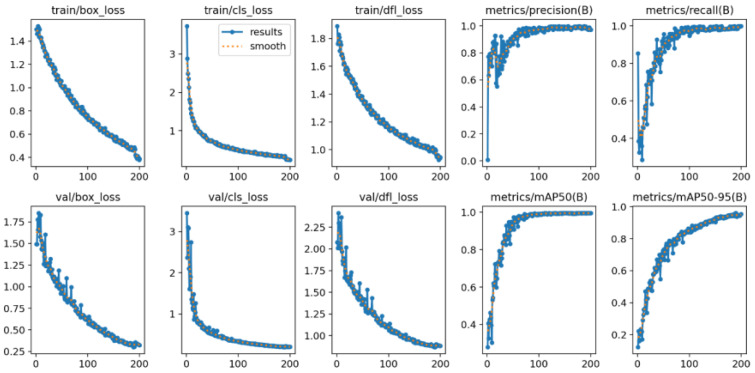
Iteration loss situation.

The proposed model demonstrates robust learning capability and excellent generalization performance in manhole cover defect detection. As shown in Figure 12, the training and validation losses rapidly converged within the first 100 epochs, with the final validation loss stabilizing at 0.095. This rapid convergence indicates that the model efficiently learned the key features of manhole cover defects during the training phase. The low validation loss also suggests that the model has strong generalization ability, capable of accurately predicting unseen data. This is crucial for real-world applications where the model must handle diverse and complex scenarios.

**Figure 13:**
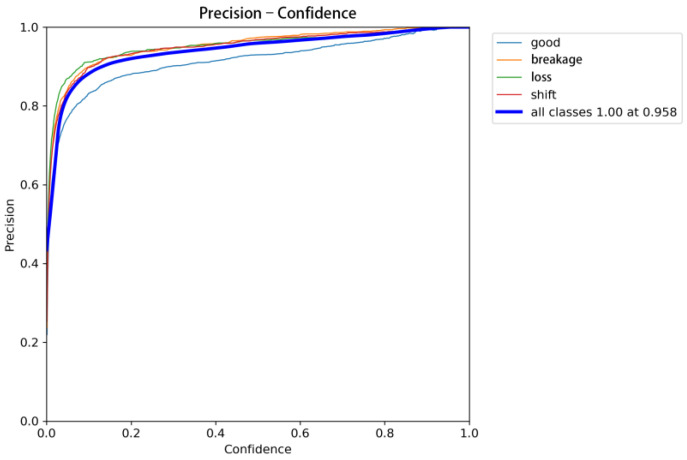
Precision–confidence curve.

The model’s classification performance was evaluated using metrics such as the mean average precision (mAP), precision, recall, and F1-score. As shown in Figure 13, at a confidence threshold of 0.5, the model achieved an mAP of 0.974 and a recall of 0.974. These high values indicate that the model can effectively detect and classify manhole cover defects across various categories. Specifically, the precision reached 0.964 for the “good” category, 0.982 for the “breakage” category, and 0.976 for both the “loss” and “shift” categories. As the confidence level increased, the precision gradually stabilized and remained high in the high-confidence region, achieving perfect precision (1.00) at a confidence level of 0.958. This suggests that the model has a strong ability to distinguish between different types of defects with high confidence, even in challenging scenarios.

**Figure 14:**
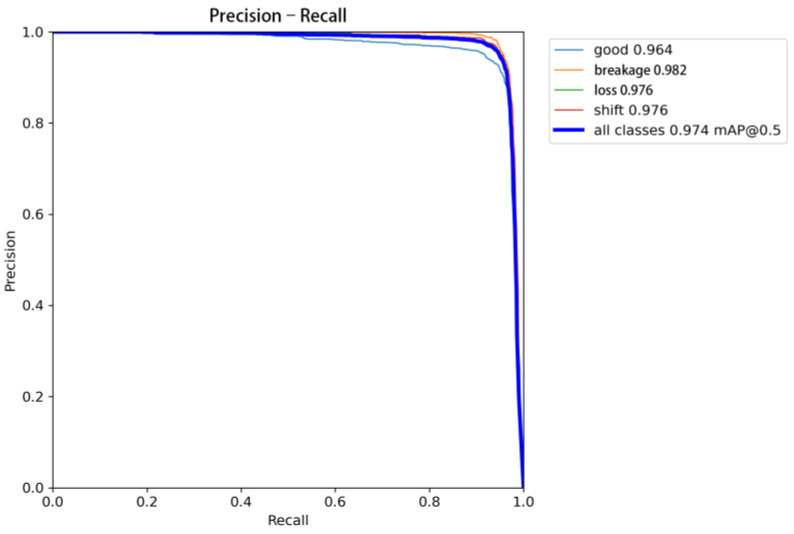
Precision–recall curve.

Figure 14 further illustrates the model’s balance between precision and recall. The F1-score, which is the harmonic mean of precision and recall, reached 0.95 at the optimal confidence level. This high F1-score indicates that the model effectively balances the trade-off between precision and recall, ensuring both high accuracy and low false-negative rates. This balance is particularly important for manhole cover defect detection, where missing a defect (false negative) can have significant safety implications.

**Figure 15:**
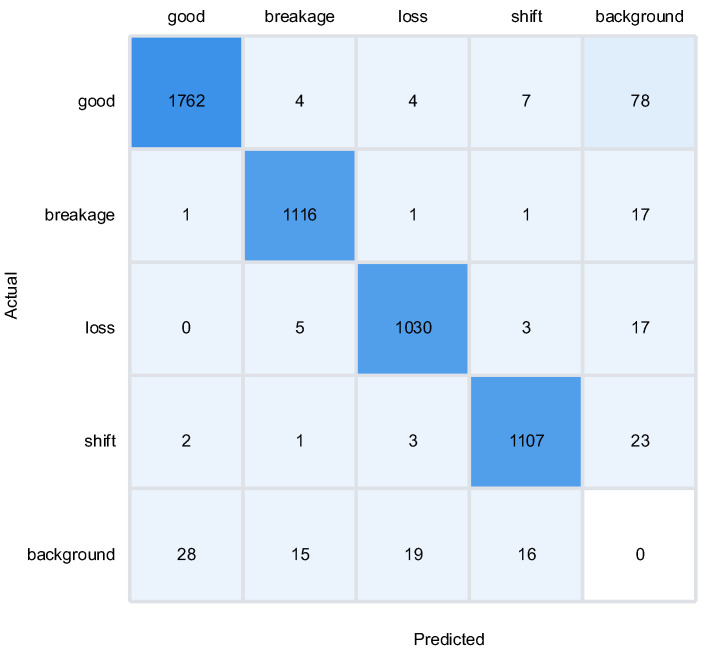
Confusion matrix of classification results.

The confusion matrix analysis (Figure 15) provides a detailed breakdown of the model’s classification performance across different defect categories. The model achieved a classification accuracy exceeding 95% for all categories, with only minor cross-misjudgment (approximately 3.5%) observed between the “breakage” and “loss” classes. This cross-misjudgment is likely attributable to the similarity in some of their geometric and visual features, such as small cracks or deformations that may appear similar in certain lighting conditions. However, the overall high accuracy (98.6%) demonstrates that the model can effectively distinguish between different types of defects, even in complex scenarios with varying lighting conditions and background interference.

**Figure 16:**
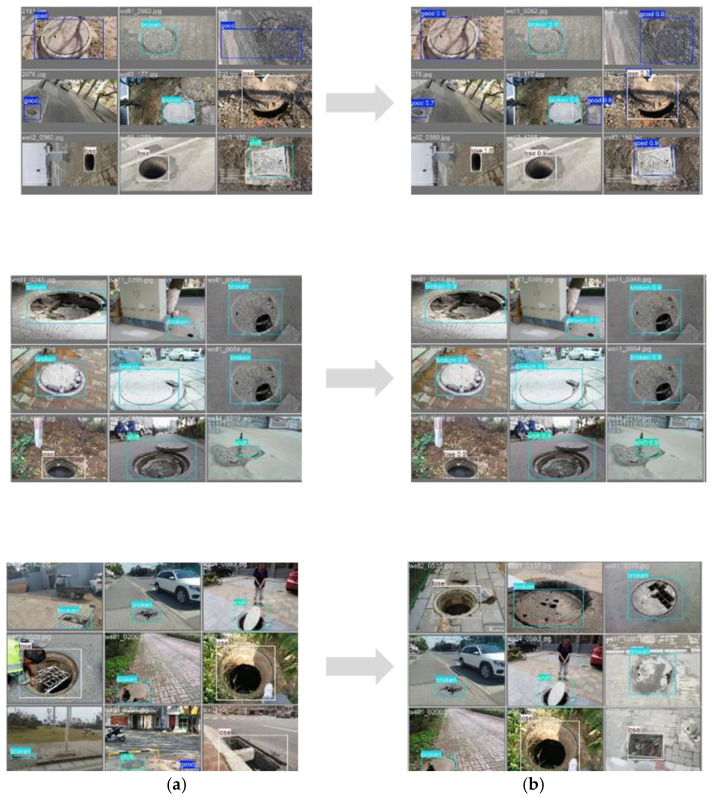
Comparison of model predictions and true labels: (**a**) True labels. (**b**) Prediction results.

To further visualize the model’s performance, Figure 16 compares the true labels and prediction results of the manhole cover defect images. The results show that the model can accurately identify and classify different types of defects, even in complex scenarios with varying lighting conditions and background interference. The high consistency between the true labels and prediction results confirms the model’s robustness and reliability for practical applications. This level of accuracy and generalization ability makes the proposed model highly suitable for real-time manhole cover defect detection in urban environments.

#### 5.3.2. Ablation Study

**Figure 17:**
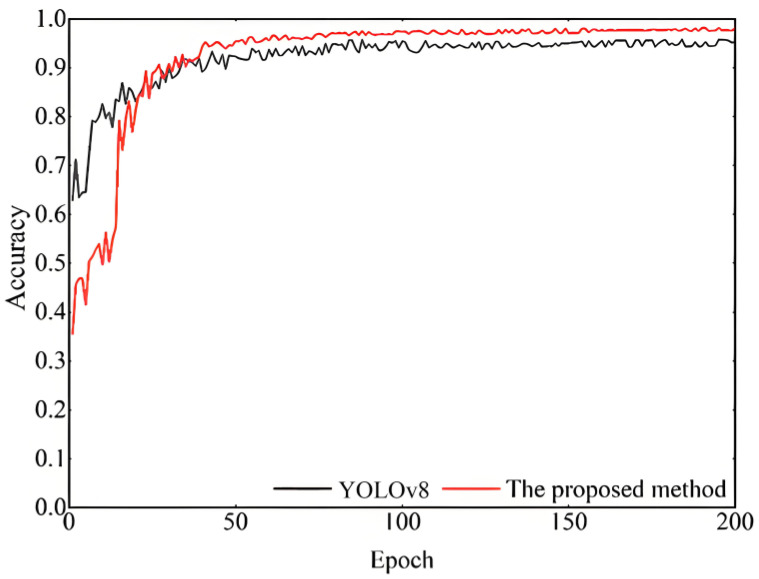
Comparison of accuracy before and after improvements in YOLOv8.

**Figure 18:**
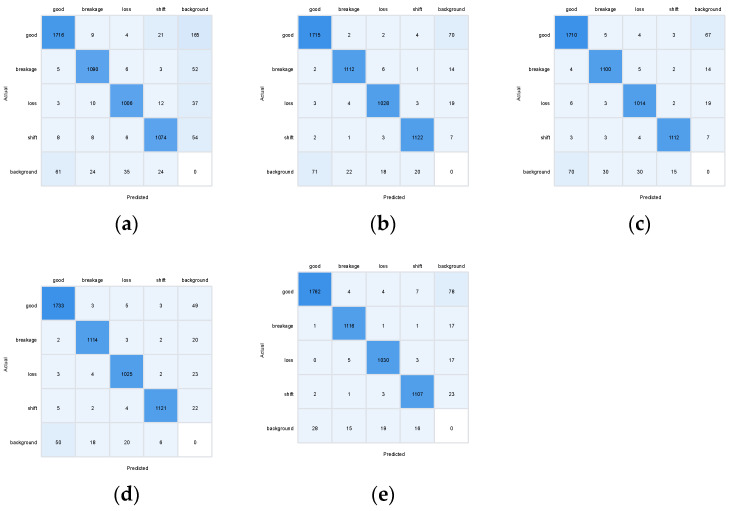
The confusion matrices of different model variants. (**a**) YOLOv8-Base. (**b**) YOLOv8-OAN. (**c**) YOLOv8-EN. (**d**) YOLOv8-EA. (**e**) YOLOv8-EAN.

This study employed YOLOv8 as the baseline model and systematically validated the effectiveness of the proposed improvements through ablation experiments. Specifically, this paper conducted a comprehensive evaluation of the EfficientNetV2 backbone network, the Outlook Attention mechanism, and the NAS-FPN feature fusion module. Figure 17 illustrates the accuracy trends of the improved model compared to the original YOLOv8 during the training process. From the curves, it can be observed that the improved model (red line) exhibited faster convergence in the early stages of training and achieved significantly higher stable accuracy after the 50th epoch compared to the baseline model (black line). Through confusion matrix analysis (Figure 18a–e), it was found that the original YOLOv8 demonstrated accurate recognition of the “good” class (1736 instances) but suffered from false positives in defect detection. The introduction of EfficientNetV2 improved the recognition of the “good” class (1755 instances) and reduced false positives. The incorporation of Outlook Attention enhanced the detection of challenging defects, while NAS-FPN optimized inter-class discrimination. Ultimately, the combination of all three modules significantly improved the recognition accuracy for each class (1762, 1808, 1760, and 1787 instances, respectively) and reduced inter-class confusion, thereby validating the performance advantages of the improved model.

To comprehensively evaluate the model’s performance, this study employed four metrics for quantitative analysis: accuracy, recall, F1-score, and precision. As shown in Table 3, when the EfficientNetV2 backbone network was introduced, the model’s accuracy increased by 1.1 percentage points (from 0.951 to 0.962). With the addition of the Outlook Attention module, the accuracy further improved by 1.92 percentage points (from 0.962 to 0.9812). When the NAS-FPN structure replaced the original feature fusion network, the accuracy increased by 1.6 percentage points compared to that of the baseline (from 0.951 to 0.967). These results fully demonstrate the effectiveness of each improvement module in enhancing model performance.

Based on the single-module validation results, this study further explored the synergistic effects of multi-module combinations. When EfficientNetV2, Outlook Attention, and NAS-FPN were integrated simultaneously into the YOLOv8 network, the model’s performance was significantly improved: accuracy reached 0.9812 (an increase of 3.02 percentage points), recall reached 0.97095 (an increase of 3.045 percentage points), F1-score reached 0.9794 (an increase of 2.88 percentage points), precision reached 0.98796 (an increase of 2.706 percentage points), and Inference Time on RTX 4090 (ms) reached 9.8 (a decrease of 3.0 ms). These results indicate a clear complementary effect among the three improvement modules, collectively enhancing the model’s performance. Specifically, the lightweight nature of EfficientNetV2 provides efficient feature extraction capabilities, the Outlook Attention module enhances the model’s perception of critical features, and NAS-FPN optimizes the fusion of multi-scale features. This multi-module synergy not only improves the overall detection performance but also maintains low computational overhead, achieving a good balance between detection accuracy and model complexity.

In order to explore the impact of different backbone networks on the detection performance of YOLOv8 model, the backbone replacement experiment of the system was carried out, as shown in Table 4. YOLOv8 base, the backbone network of YOLOv8, was replaced by ZResNet50, MobileNetV3, DenseNet121 and EfficientNetV2 backbone networks and trained and tested under the same dataset and experimental environment. The experimental results show that the model using efficientnetv2 as the backbone performed best in all detection accuracy indexes, and its accuracy rate of 0.965, accuracy rate of 0.962, recall rate of 0.951, and F1-score of 0.957 were significantly better than those of the original YOLOv8 base model and other alternatives. Its reasoning time of 7.3 ms was also lower than that of the base model of 8.5 ms. It is worth noting that the model using lightweight MobileNetv3 showed the fastest reasoning speed of 4.8 ms, but with the decline in detection accuracy, it is considered that EfficientNetv2 showed the best comprehensive performance under the experimental setting.

#### 5.3.3. Comparison of Experiments with Different Models

**Figure 19:**
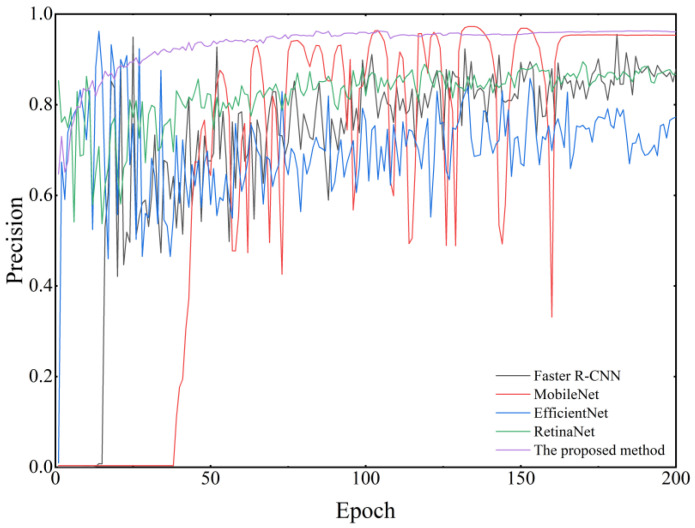
Comparison of accuracy among different models.

Compared to other models, the improved YOLOv8 model demonstrates superior performance in terms of detection accuracy, parameter count, and computational cost [45,46,47,48,49]. As shown in Figure 19 and Table 5, the model achieves an mAP@0.5 of 98.12%, an mAP@[0.5–0.95] of 97.10%, and an F1-score of 97.94%. Meanwhile, the model has only 2.12 M parameters and a computational cost of 7.3 GFLOPs. Compared to MobileNet, the improved YOLOv8 model shows increases of 6.76%, 6.40%, and 6.38% in mAP@0.5, mAP@[0.5–0.95], and F1-score, respectively, while reducing the parameter count by 49.88% and the computational cost by 87.93%. Compared to Faster R-CNN, the improved YOLOv8 model achieves improvements of 10.25%, 10.60%, and 10.70% in the same metrics, with a reduction of 93.85% in parameter count and 93.92% in computational cost. When compared to EfficientNet, the improved YOLOv8 model shows enhancements of 6.85%, 6.25%, and 6.37% in these metrics, while decreasing the parameter count by 72.82% and the computational cost by 82.50%. Compared to RetinaNet, the improved YOLOv8 model achieves gains of 12.44%, 12.10%, and 12.20% in mAP@0.5, mAP@[0.5–0.95], and F1-score, respectively, with a 94.85% reduction in parameter count and a 92.70% reduction in computational cost. The influence time on RTX 4090 (ms) is only 9.8 ms, far ahead of other models. In summary, the improved YOLOv8 model achieves a high level of detection accuracy while maintaining low model complexity, effectively balancing detection performance and model lightweightness.

## 6. Case Study

**Figure 20:**
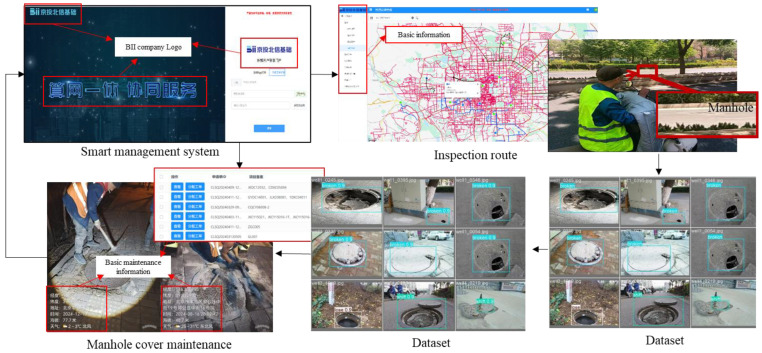
Management process of intelligent operation and maintenance system.

Beijing Information Infrastructure Construction Co., Ltd. (BII) is mainly responsible for the operation, maintenance, and management of underground communication pipelines in the urban area of Beijing. With the rapid development of the city, there is an urgent need to improve management efficiency. BII has upgraded its management system mainly through the visualization of the management system and the empowerment of the intelligent identification system for manhole cover defects. Based on the current management content of the company, BII has established a smart management system, which is divided into three modules, as shown in Figure 20.

1. Inspection module: This includes the setting of daily inspection routes, task allocation, and inspection personnel information. Real-time inspection personnel positions can be seen in the system. Inspection personnel upload manhole cover images to the system by taking photos. The inspection routes are updated on a weekly basis based on last week’s manhole cover defects and repair locations.
2. Manhole cover defect recognition module: Embedded with a smart recognition system for manhole cover images, this classifies and recognizes defects in manhole cover images uploaded during inspections, filters out and reports pictures with defects, and regularly uploads manhole cover images uploaded during inspections to the manhole cover health image database.
3. Maintenance work order distribution module: This reviews the accuracy of identified defect images, determines the defect repair plan, and determines the work order distribution time and maintenance cycle based on the urgency of the manhole cover defect.

Through the above modules, the rapid and visual management of manhole cover defects is achieved as a whole. The traditional inspection–reporting–dispatching–inspection–maintenance cycle is optimized to the inspection–defect self-identification–dispatching–maintenance cycle, greatly improving management efficiency. The management efficiency is increased from 48 h in two inspection cycles to 24 h in one inspection cycle.

The modular design of the intelligent operation system is transferable to other urban defect detection tasks, such as pothole identification, graffiti detection, or streetlight malfunction monitoring. From the perspective of urban planning, it will greatly improve the current urban infrastructure operation and maintenance efficiency. It will focus more on saving time and space, reducing manpower, improving the intelligent level of operation and maintenance, improving urban capacity, and reducing the pressure of operation and maintenance management.

## 7. Conclusions

Aiming to solve the problems of low feature extraction efficiency, insufficient multi-scale feature fusion, and the difficulty in balancing model accuracy and lightweightness in manhole cover defect detection, this paper proposes an intelligent recognition model for manhole cover defects based on the improved YOLOv8. By introducing the EfficientNetV2 lightweight backbone network, the Outlook Attention mechanism, and the NAS-FPN feature fusion architecture, the model’s feature extraction capability and multi-scale feature expression ability are significantly enhanced. Moreover, the hybrid loss function design, which combines GIoU loss, dynamic weighted BCE loss, and Distribution Focal Loss (DFL), effectively mitigates the impact of sample imbalance and inter-class differences on model performance. Experimental results show that the improved model achieves an overall recognition accuracy of 98.6% on the manhole cover defect dataset, with an mAP@0.5 of 98.12%. The model has only 2.12 M parameters and a computational cost of 7.3 GFLOPs, achieving an excellent balance between detection accuracy and lightweightness. Ablation experiments and comparative analyses further validate the effectiveness of each improved module. Compared with mainstream models such as MobileNet and Faster R-CNN, our model demonstrates significant advantages in terms of accuracy, parameter count, and computational efficiency. Additionally, the proposed model serves as a core component of an intelligent operation and maintenance management system for urban manhole covers, which integrates perception, identification, warning, and response into a closed-loop framework, thereby significantly enhancing the real-time response capability and reliability of urban infrastructure management. This study has proven the effectiveness of EffectiveNetv2 in the field of urban manhole cover defect identification. The proposed lightweight detection framework and closed-loop management system offer a replicable blueprint for the real-time monitoring of diverse urban defects, advancing the resilience of smart cities. From the perspective of urban planning, the automatic detection method can be extended to the operation and maintenance management of defects in multiple scenes in cities, greatly improving management efficiency, and improving technical support for promoting the construction of smart cities and the intelligent transformation of infrastructure operation and maintenance management.

## Figures and Tables

**Table 1:** Environment configuration.

Environment Item	Configuration
CPU	Intel Core i9-13900K
GPU	NVIDIA RTX 4090
Memory	64 GB DDR5
Operating System	Ubuntu 20.04 LTS
Python Version	Python 3.11.5
CUDA Version	CUDA 12.1
Pytorch Version	Pytorch 2.1

**Table 2:** Experiment configuration.

Item	Configuration
Optimizer	AdamW
Learning Rate	0.001
Batch Size	64
Training Epochs	200
Early Stopping Patience	100
Image Dimensions	512 × 512

**Table 3:** Ablation experiment data table.

Model	YOLOv8	EfficientNetV2	Outlook Attention	NAS-FPN	Accuracy	Precision	Recall	F1-Score	Inference Time on RTX 4090 (ms)
YOLOv8-Base	√				0.951	0.9609	0.9405	0.9506	8.5
YOLOv8-OAN	√		√	√	0.962	0.97	0.954	0.973	12.8
YOLOv8-EN	√	√		√	0.967	0.963	0.954	0.965	7.2
YOLOv8-EA	√	√	√		0.965	0.975	0.96	0.963	8.9
YOLOv8-EAN	√	√	√	√	0.9812	0.98796	0.97095	0.9794	9.8

**Table 4:** Detection effects of different backbone networks.

Model	Accuracy	Precision	Recall	F1-Score	Inference Time on RTX 4090 (ms)
YOLOv8-Base	0.951	0.9609	0.9405	0.9506	8.5
YOLOv8-ResNet50	0.923	0.925	0.900	0.912	15.2
YOLOv8-MobileNetV3	0.895	0.898	0.883	0.890	4.8
YOLOv8-DenseNet121	0.908	0.910	0.895	0.902	11.5
YOLOv8-EfficientNetV2	0.965	0.962	0.951	0.957	7.3

**Table 5:** The comparison table of experimental data from different models.

Model	mAP@0.5/%	mAP@[0.5–0.95]/%	F1-Score/%	Params/M	GFLOPs	Inference Time on RTX 4090 (ms)
MobileNet	91.36	90.7	91.56	4.23	5.8	8.2
Faster R-CNN	87.87	86.5	87.24	34.5	120	125.8
EfficientNet	91.27	90.85	91.57	7.8	4.0	12.5
RetinaNet	85.68	85	85.74	41.2	100	115.4
Improved YOLOv8	98.12	97.10	97.94	2.12	7.3	9.8

## Data Availability

Data will be made available on request.
